# Supplementary material for: Are carbon emissions trading and green financial instruments synergistic? -Comprehensive quantitative research based on content analysis
Source: PLoS One. 2024 Mar 7;19(3):e0298601. doi: 10.1371/journal.pone.0298601 (PMC10919601; doi:10.1371/journal.pone.0298601)
Supplement: S1 Data — (ZIP) [file pone.0298601.s001.zip › Data/地级市Z_F工作报告.zip!/╜¡╦╒/╤∩╓▌╩╨/└┤╫╘╣1⁄2╓┌║┼í╛┬φ┐╦╩2╛▌═°í┐_2013.docx]

各位代表：

现在，我代表扬州市人民政府向大会作工作报告，请予审议，并请市政协各位委员和其他列席人员提出意见。

2012年工作回顾

2012年，面对国际国内经济发展环境复杂困难的形势，我们在中共扬州市委的领导下，团结和依靠全市人民，以科学发展观为统领，围绕市六届人大五次会议和七届人大一次会议确定的目标任务，克难求进，开拓创新，扎实推进“三个扬州”和世界名城建设，取得了良好成效。预计全市实现地区生产总值2950亿元，增长11.5%。财政总收入554.51亿元，公共财政预算收入225亿元；税收收入180.61亿元，增长16.2%；公共财政预算收入税收占比80.3%，比上年提高9个百分点。城市居民人均可支配收入28370元，增长14.5%；农民人均纯收入12900元，增长15%。

加强分析研判，努力把握主动权，经济保持平稳增长。一是狠抓项目建设。通过加强要素保障、协调服务、督查考核，推进实施了一批重大产业项目和基础设施项目。上海大众30万辆整车等30个重大项目投产。普洛斯物流等100个重大项目开工。扬州泰州机场建成通航。江都至六合高速公路、333省道高邮西段和新民滩特大桥建成通车。扬州港新增万吨级泊位5座。润扬河工程建成通水，南水北调东线一期工程扬州段基本建成，淮河入江水道整治工程加快建设。500千伏仪征输变电工程建成运行。固定资产投资增长20.3%。二是着力抓好内需。在巩固零售、餐饮等传统消费市场的同时，活跃汽车、家电等消费市场，社会消费品零售总额963亿元，增长14.3%。采取有效措施，促进房地产业稳定健康发展，完成投资220亿元，竣工商品房480万平方米。出台促进和支持建筑业发展意见，实现建筑业总产值2100亿元，增长15%。三是推进经济国际化。举办“烟花三月”国际经贸旅游节和名城扬州携手世界名企联谊会等境内外招商活动，注册外资实际到账21亿美元，新引进世界500强企业10家。促进外贸企业调优产品结构、加强品牌建设、开拓新市场，实现进出口总额102亿美元，其中出口80亿美元，增长9.3%。完成外经营业额4.1亿美元，增长17.3%。四是全力支持企业发展。召开全市企业发展大会，开展“走进企业、服务企业”系列活动，在土地供应、融资担保、成果转化、人才员工、市场开拓等方面抓好服务。推进与央企的合作。帮助企业向国家和省争取各类财政扶持资金7.6亿元、债券融资45.2亿元，推动银企签约53项、发放贷款256亿元。分别出台支持国资公司优化发展的意见。新增民营企业注册资本金实际到资430亿元，增长22.8%。

加快结构调整，推进科技创新，转型发展步伐坚定。一是先进制造业健康发展。重点围绕五大千亿级产业分别研究发展战略和举措。落实国家各项扶持政策，加大对新兴产业金融和人才支持。机械装备、汽车及零部件、船舶及配套件、石油化工产业实现产值4830亿元，增长14.5%；新能源、新光源产业实现产值600亿元，增长17.5%。二是现代服务业加快发展。编制文化创意、工业设计、科技金融等新兴服务业发展规划。制定扶持政策，推进服务业九大产业和地区总部经济、会展业加快发展。实现服务业增加值1180亿元，增长12%；占地区生产总值比重40%，比上年提高1.3个百分点。旅游业总收入430亿元，增长16%。软件与信息服务业业务收入185亿元，增长41.7%。年末人民币存贷款余额3273亿元和2020亿元，分别增长16.1%、17.7%。三是提高科技创新能力。西安交大扬州科技园建成开园，东南大学扬州科技园和研究院启动建设。获批江都建材装备、仪征汽车及零部件、高邮特种电缆3个省级科技产业园。新增国家高新技术企业58家，获批省级以上两化融合示范试点企业36家。省级以上高新技术孵化器孵化面积64.6万平方米、在孵企业627家。全社会研发投入占地区生产总值比重达到2.1%，实现高新技术产业产值3200亿元，占全市规模以上工业总产值比重43%。新增专利授权7571件、发明专利授权443件，分别增长56%和75%。创成中国驰名商标5件。扬州被分别授予“中国城市信息化推进”和“中国智慧城市推进”十强称号。四是推进园区提档升级。出台关于推动扬州经济技术开发区跨越发展和促进市化工园区绿色发展的意见。专题研究各省级开发园区发展定位，实行一园一策。6家省级以上开发区进入全省第一板块，扬州经济技术开发区获批国家科技兴贸创新基地，邗江开发区创成省高新技术开发区，广陵产业园升格为省级开发区。全市开发园区业务总收入、工业产品销售收入、公共财政预算收入分别增长36%、36%、30%。五是强化节能减排。全年实施节能技术改造项目127项、循环经济项目39项、减排项目171项，淘汰落后用能设备2232台（套），关闭“五小”企业104家。全市单位地区生产总值能耗下降4%，实现节能34.6万吨标准煤，节能减排和重金属污染综合防治工作完成省定目标。

抓好城乡统筹，推动融合发展，城乡一体化进程加快。一是推动沿江地区率先融合发展。实施沿江地区融合发展行动计划，优化重大基础设施和产业布局，推进沿江各市（区）组团之间及沿江各区域内部的融合。谋划和推动江广融合地带建设。新城西区城市副中心功能进一步完善。二是提升主城区建设和管理水平。推进瘦西湖隧道等重大基础设施建设，友谊路拓宽改造竣工通车，文昌路东延及广陵大桥、仙女大桥建成。推进“清水活水、不淹不涝”城市建设，完成河道生态清淤6条，整治积水路段14个。推进古城保护，东关历史文化街区通过4A级景区验收。

国展中心二期建成，西部客运枢纽开工。开展占道经营、违法建设、渣土车等专项整治行动，全面提高城市管理水平。开发利用地热资源，扬州被国土部命名为“中国温泉之城”。城镇化工作稳步推进，全市城镇化率59.4%。三是积极推进“三农”工作。落实各项惠农强农政策。全年粮食总产306.4万吨，实现“九连增”。新增设施农业12.3万亩、设施渔业5.4万亩，新增高标准农田15万亩。县级以上农业龙头企业销售收入、利润分别增长20.6%、15.7%。工商登记的农民专业合作社累计3464个。村级“四有一责”建设成效明显，扶持经济薄弱村建设标准厂房累计95万平方米。承办第14届江苏农业国际合作洽谈会。新建市区“菜篮子”基地2500亩。农机、粮食、供销、气象、农业科研在“三农”工作中发挥重要作用。四是壮大县域经济。实施沿江地区率先基本实现现代化和沿河地区加速崛起行动计划。争取省财政提高对高邮宝应转移支付比例、扶持南北园区共建政策和城镇化建设试点取得突破。推进万顷良田建设工程试点，完成土地整治4万亩。五是加强生态环境建设。全市新增造林8.81万亩，市区新增绿地125.48万平方米。市区空气质量优良天数322天，城市饮用水源地水质达标率100%。实施“美好城乡建设行动”，继续开展农村河道河塘疏浚整治，完成“六路一边”村庄整治任务，新创省三星级“康居乡村”60个、市级“优美乡村”10个。生态市创建通过国家级考核验收。

积极深化改革，创新社会管理，和谐社会建设成效明显。一是协调推进各项改革。事业单位清理规范工作全面完成，绩效工资改革稳步实施。公立医院改革试点和规范化建设有序进行，医改主要工作任务和基层医疗卫生机构债务化解工作基本完成，乡村卫生机构一体化管理基本实现。做好“营改增”试点工作。高邮市和杭集镇、邵伯镇入选第三批全国发展改革试点城镇。二是发展各项社会事业。创成江苏省教育现代化建设先进县（市）区2个。南邮通达学院来扬办学。育才小学东区校建成招生。创成省四星高中2所、省优质幼儿园13所。新招宏志班24个。中等职业教育创成省品牌和特色专业9个。高考本二上线人数增长11.5%。社区卫生服务中心（乡镇卫生院）标准化达标率97.1%。实行新型农村合作医疗补偿“十个统一”政策，参合农民政策范围内住院补偿比例达75%。健全公共文化服务体系，新建村级文化广场300个，文化惠民活动深入开展。曲艺中篇《盛世红伶》获全国“牡丹奖”，木偶剧《琼花仙子》获国际木偶节最佳剧目奖。纪录片《自然之子》获中国广播电视星光奖。成功举办世界运河名城博览会暨世界运河大会。新实施文化博览城项目7个。大运河申遗文本预提交世界遗产中心。非遗保护工作成效明显。推进全民健身服务体系建设，扬州马拉松赛被国际田联授予“金标赛事”称号。人口计生、科普、档案、方志、台湾事务、侨务、外事和工会、青年、妇女、儿童等事业取得新进步。三是加强和创新社会管理。推进和谐社区建设，建成社区工作站282个，市区社区服务中心建设覆盖率86.6%。深化行业协会和社会管理改革创新，出台政府购买社会组织服务政策意见和实施办法。开展领导干部接待群众来访工作，办结信访积案276件。全市各类调解组织受理矛盾纠纷2.2万件，调处成功率98.6%。市级民主法治村（社区）建成率95%。做好“四项排查”工作，加强社会稳定风险评估、情报信息研判和网上舆情导控。开展治安重点地区整治，公众安全感继续位居全省前列。实施食品药品生产流通领域集中整治行动，着力解决影响群众饮食用药安全的突出问题。严格落实安全生产责任制，加强对交通运输、消防、建筑施工和危险化学品等重点领域的安全监管。迅速处置、科学应对高邮、宝应地区“7·20”地震，救灾和灾后恢复工作有力有效。平安扬州和法治扬州建设取得新进展。国防动员和后备力量建设取得新成效，实现全国双拥模范城“六连冠”。民族、宗教、民防、仲裁等工作扎实开展。

致力增加居民收入，切实为民排忧解难，民生工作扎实有效。一是实施城乡居民收入倍增计划。多措并举建设充分就业城市，新增城镇就业6.1万人，转移农村劳动力1.4万人，城镇登记失业率2.4%。“就业e图”获工信部创新应用奖。开展创业培训1.1万人，新建创业孵化基地15个。完善工资正常增长机制和工资集体协商制度。引导鼓励城乡居民拓宽投资渠道，城镇家庭经营性、财产性收入分别增长15%、8%，农村家庭经营性、财产性收入分别增长10%、25%。实施了城乡低保、农村五保、孤儿赡养标准的新一轮提标。二是提升社会保障水平。企业职工养老保险净增缴费4.5万人，城乡居民基本养老保险参保覆盖率98.5%，被征地农民社会养老保险覆盖率96.4%。落实提高企业退休人员养老金政策。城镇职工和城镇居民医保政策范围内住院支付比例分别达82%、70%。推进市区社会保险征缴政策、待遇水平、信息系统的统一。三是推进民生工程。保持物价基本稳定，居民消费价格指数103%。建设平价商店75个，发放困难群体价格补贴1492.53万元。如期实现区域供水全覆盖。新改建农村公路230公里、危桥100座。基本完成农产品市场检测室升级改造。市食品安全快速检测中心建成启用。市区整治改造老小区40个、“城中村”15个，完成公有住房解危6.18万平方米，调整优化公交线路13条。全市新建经济适用房2000套、公租房7491套（间）、廉租房360套。住房公积金新增扩面7.5万人。新建社区（村）居家养老服务中心157个。扬州被评为全国公益慈善七星级城市。老龄、残疾人事业取得新业绩。

强化能力作风建设，努力提高服务水平，政府自身建设进一步加强。继续开展“三下三联三交”活动。围绕事关经济社会发展和民生改善等6项重点课题进行深入调研。认真接受市人大及其常委会的法律监督、工作监督和市政协的民主监督，广泛听取各民主党派、工商联、无党派人士和社会各界意见。全年办理市人大代表议案、建议472件，政协提案596件。科学界分权力，归还和下放给广陵区459项行政事权。畅通重大项目行政审批“绿色通道”，实施基本建设项目并联审批改革。加强政务公开和新闻发布工作，完成“中国扬州”门户网站群主站和95个子站整合建设。政府信息公开满意度全省第一。开展领导干部到12345政府服务热线中心现场接听群众来电活动，回复率、解决率均达94%以上。制定政府规范性文件18件。办理行政复议案件354件。开展“三公开、三报告”电视直播活动。实施重大项目监督检查和考核问责办法。强化行政效能监察和审计监督，开展工程建设等领域突出问题专项整治，查处了672件违纪违法案件。

各位代表，这些工作的开展和成绩的取得，是中共扬州市委正确领导的结果，是市人大、市政协有效监督、大力支持的结果，是全市人民攻坚克难、共同奋斗的结果。在此，我代表扬州市人民政府，向全市人民，向人大代表、政协委员，向各民主党派、工商联、无党派人士、各人民团体，向驻扬部队、驻扬单位，向所有关心、支持、参与扬州建设和发展的同志们、朋友们，表示崇高的敬意和衷心的感谢！

在肯定工作和成绩的同时，我们也清醒地看到经济社会发展中还存在一些深层次的矛盾和问题，主要是：做大经济总量和提升发展质量的双重要求十分迫切；部分行业经济下行和资源环境制约的双重压力持续加大；城乡居民收入稳定增长和维护社会和谐稳定的双重任务依然繁重；做大做强县域经济和推进城乡统筹发展的双重目标难度较大；政府行政管理和公共服务的双重能力还需不断提高等等。对此，我们一定高度重视，并采取切实有效措施加以解决。

2013年工作安排

2013年政府工作的总体要求是：高举中国特色社会主义伟大旗帜，以邓小平理论、“三个代表”重要思想、科学发展观为指导，全面贯彻落实党的十八大和中央、省经济工作会议精神，按照市委六届四次全会的部署和要求，稳中求进、进字当先，强化基本、扎实开局，进一步推进创新转型，进一步深化改革开放，进一步促进城乡统筹，进一步加强社会建设，进一步提升生态文明，进一步增进民生幸福，全力推进“三个扬州”和世界名城建设，加快建成更高水平小康社会。

2013年全市经济和社会发展的主要预期目标为：在提高质量和效益的基础上，地区生产总值增长11%。公共财政预算收入增长8%。固定资产投资增长20%。社会消费品零售总额增长14%。城市居民人均可支配收入和农民人均纯收入增幅高于省均水平。城镇登记失业率控制在4%以内。节能减排完成省下达任务。

重点做好以下八个方面工作：

一、以稳中求进为首要任务，保持经济持续健康发展

抓好工业重大项目建设。全力抓好一批新的重大项目，新开工10亿元以上工业重大项目45个。制定出台项目绩效评估办法和按项目质量供应资源要素的政策措施，努力提高项目质量和产出效益。推动一批项目投产达效。加快潍柴亚星汽车等16个重大项目建设进度，确保年内竣工投产；抓好已竣工的上海大众整车等20个重大项目尽快达产达效。推动一批中小型技改示范项目实施。加快产业技术进步，出台支持企业技术改造政策意见，实施亿元以上技改项目150项、重点技改示范项目20项、重点成果转化项目20项。在突出抓好工业重大项目的同时，继续扩大全社会有效投入，改善投资结构，完成全社会固定资产投资2000亿元以上。

扶持实体经济发展。制定并落实推进基本产业发展的具体举措。出台扶持本土企业和培育优秀企业家意见，对重点行业和骨干企业实行一业一策、一企一策服务。高度重视建立小微企业服务体系，推动小微企业“专、精、特、新”发展。开展金融服务实体经济十项行动，工业贷款增速高于全省平均水平。进一步完善金融服务市场，引进汇丰银行，设立村镇银行、小额信贷公司10家以上。大力推进企业上市和直接债务融资工具的运用。帮助企业解决用工矛盾，支持企业培养和引进高管人才、技术骨干。规模以上工业产值增长15%。

发展现代服务业。围绕促进经济发展、体现精致扬州特色、提高市民生活品质，着力优化服务业布局，提升服务业发展层次，做大做强服务业。确保服务业增加值占地区生产总值比重提高1个百分点。市级以上服务业集聚区年营业总收入增长15%。拓展提升旅游业。推进文化与旅游深度融合，发展商务旅游；强化县域特色旅游项目规划和培育；围绕实现旅游“淡季不淡”，规划建设具有温泉酒店特色的旅游度假区，全年旅游业总收入突破500亿元。加快发展现代物流业。以“亿吨大港、百万标箱”为目标，整合沿江、沿河港口资源，实施江河联动，打造区域性物流中心；规划建设空港产业园，争取扬州泰州机场实现一类航空口岸开放；建设扬州综合保税区并争取年内获批。大力发展以办公室为载体的软件和信息服务业，以实验室为载体的研发产业，以工作室为载体的文化创意产业。细化落实特别机制和特殊政策，培育发展地区总部经济和会展经济。

增强消费拉动作用。巩固传统消费市场，积极开拓中介服务业、高新技术服务业等新领域，积极培育文化、教育、康体、家居服务等新热点，积极发展电子商务、网络购物等新业态。建设广陵区、江都区、邗江区四个专业批发市场，加快万科商业综合体、扬州商城综合体、京华城综合体等建设进度。推进“万村千乡市场工程”，提升农家店建设质量和物流配送水平。加强产品质量监管，营造放心消费、安全消费环境。推动住房产业转型升级，促进房地产市场健康发展。

二、以创新转型为核心，加快提升产业竞争力

增强企业自主创新能力。突出企业创新主体地位，加快提升企业集成创新和引进消化吸收再创新能力，运用高新技术和先进适用技术改造提升现有产业，新建省级以上“三站三中心”40家，培育科技型拟上市企业10家、高新技术企业60家、科技创新示范企业100家、民营科技企业1000家，鼓励企业创建国家级研发机构，实现大中型工业企业研发机构全覆盖。继续实施“上市、专利、标准、品牌”四大战略和“千企创新升级”计划，设立市知识产权维权援助服务中心，新增注册商标3500件、省级以上品牌30件以上。

推进创新创业平台建设。开展国家级创新城市建设。启动规划建设科教创新城。全面推动主城三区和扬州经济技术开发区20万平方米，宝应、高邮、仪征10万平方米以上具有专业和特色的科技与创新产业综合体建设。启动建设国际技术转移及科技成果转化中心。以创业融资、居住奖励、子女教育等政策创新为核心，持续优化人才发展综合环境，引进国内外知名企业研发中心和科技职业经理人团队入场，吸引国内外知名高校院所的技术和成果来扬转化。加大招才引智力度，全年引进创新创业领军人才100人。加强对本土优秀人才的培养，发展一批高技能人才培养基地、技能大师工作室，进一步提高技能人才占技能劳动者的比重。

深化政产学研金合作。组织和推动企业向国家争取牵头实施产业目标明确的重大科技项目。提升南大—扬州光电研究院、南大—扬州化工研究院、西安交大扬州科技园等研发平台的建设和服务水平。推进与中科院、中关村等共建科技园或研究院。制定并实施“科教合作新长征”计划，加强同国内外知名高等院校、科研院所的合作，高度重视与扬州大学的深度合作。新签产学研合作项目250个。加快发展科技金融，设立种子基金、天使基金，引导金融机构和社会资本对科技企业投资。

三、以增强活力为目的，扎实推进改革开放

加快开发园区建设。实施好园区特色化发展和个性化考核意见。引导各开发园区根据现有产业基础提升做大主导产业，在相关开发区和工业集中区重点做好五大千亿级产业集群。推进产城互动，科学规划布局产业、生活配套、科技创新和研发孵化器三大板块。推动园区“二次创业”，所有省级以上开发区6月底前都要实现同先进开发区的结对合作和开展产业转移对接。

提升发展开放型经济。主攻央企国企、中国百强民营企业、世界500强企业，大力实施“530”招商行动计划，继续开展“名人名企名城行”活动。办好“烟花三月”国际经贸旅游节、世界运河名城博览会、国际盆景大会等重大节庆活动。注册外资实际到账增长14%，新引进世界500强企业10家以上。扬州出口加工区用好“飞地经济”政策，新引进驻区企业20家，新增出口10亿美元。外贸出口增长15%。加快“走出去”步伐，支持企业参与境外项目投资开发，建立国际营销网络和生产加工及原料基地，外经营业额增长15%。

扶持民营经济发展。降低门槛、放宽准入，鼓励民营企业参与现代服务业、战略性新兴产业、社会公用事业建设，支持民营企业跨行业、跨地区兼并重组，促进企业经营和管理模式创新。切实帮助小微企业和个体工商户解决经营载体缺乏、融资贷款担保难等突出矛盾和问题。充分发挥商会、行业协会等组织作用。新增民资注册资本金实际到资480亿元，新开工亿元以上项目117个、10亿元以上项目20个，新增私营企业1.5万户、个体工商户2.5万户。

继续深化改革。实施职业教育资源整合。深化医药卫生体制改革。推进市区社会保险、民政福利政策的同城同步同标，逐步实现市区社会保险统筹、统一管理。整合执法管理资源，提高属地化执法管理能力，促进城市管理重心下沉。围绕加快规划落地、项目落地、开工落地，全面落实工业项目行政审批流程优化方案。取消和调整一批行政审批项目，梳理并下放一批行政事权。改革政府投资项目和现代服务业项目审批流程，积极推进基本建设项目并联审批，并试行不予行政许可汇报制、项目备案制，落实服务承诺制。积极推进街道、社区管理体制改革试点，加快实现基层社会管理“扁平化”。全面实施对国有资产保值增值考核，发挥国资公司在城市建设和社会公共服务方面的骨干作用。推进农村综合配套改革。

四、以城乡统筹为引领，全面提升城乡建设水平

扎实推进城镇化。以城镇化促进城乡发展一体化，加快城镇化步伐，提高城镇化质量，全市城镇化率达到60%。进一步完善“一带一轴”市域城镇空间布局。集聚集约整合城乡资源，推进“三集中”，有序推进农业转移人口市民化。提升县城（镇）城市功能，加快基础设施、公共服务设施向镇村延伸。加大重点中心镇投入，完善公用基础设施，促进产业发展和农村消费。新改建农村公路100公里、改造危桥100座，镇村公交开通率达40%。加强城镇就业服务体系和社会保障体系建设，推进就业、教育、医疗、养老城乡一体化。宝应、高邮成为省城镇化试点县（市）。

推进农业现代化。继续推进50亿元产值的连片农业特色产业基地建设，每个县（市）区重点打造1个省级农业园区，建成市级以上现代农业产业园区20个。新建设施农业10万亩、设施渔业5万亩。新增高标准农田15万亩。高效农业保险占农业保险比重达20%以上。推进农业产业化，培育亿元以上农产品加工龙头企业45家、10亿元以上企业8家。加快发展农业专业化服务，加强农村经纪人队伍建设，不断增强农民专业合作社的服务能力和带动能力，新创市级以上“五好”示范社100个。加强农业投入品管理。围绕农民放心种菜在田头到市民放心消费在餐桌，做好“菜篮子”工程，加大投入，减少流通环节，新扩建无公害蔬菜基地2000亩。

加快发展县域经济。落实好进一步加快县域经济发展的实施意见。完善县域功能区规划。推进工业集中区整合和提升，形成一镇一业、一镇一特色。充分发挥交通对县域经济发展的支撑作用，重点做好列入新一轮省道网规划干线公路的新建扩建，加快237省道建设，全面开工建设333省道仪征段。大力实施中央财政农田水利重点县、灌区节水改造和水利血防工程，加快水利现代化进程。积极帮助县（市）向上争取项目和资源。村级“四有一责”建设再提高，实现沿河地区行政村集体经营性收入20万元、沿江地区30万元以上。

五、以融合发展为方向，进一步做优做美中心城市

加快宁镇扬同城化步伐。推动完善宁镇扬同城化工作机制，积极推进宁镇扬资源统筹配置和宽领域全面合作。开展宁扬、扬镇城际轨道项目的前期工作。推进交通、港口、旅游、环保、社会保障等方面的深度融合。

建设重大基础设施。加强沿江地区组团间的空间联系，着力构建“三环十纵十横两个枢纽”内部交通体系。完成328国道连接线建设。推进瘦西湖隧道、新万福路、江都路北延、开发路东延、文昌路西延等工程。启动轨道交通建设规划编制工作，控制好轨道1号线、2号线、6号线站点空间。加快宁启铁路复线电气化改造。继续推进连淮扬镇铁路、沪陕高速江都至广陵段、京沪高速扩容及南延过江等一批重大基础设施项目前期工作。南水北调东线一期工程扬州段建成通水。淮河入江水道整治工程完成切滩900万立方，加固堤防55公里。实施古运河等中小河流治理工程，提高城市防洪排涝能力。推进500千伏扬州北输变电工程和配电网示范项目建设。

完善城市功能。围绕迎接扬州建城2500周年，启动实施100项城市功能性、标志性重大工程。编制“七河八岛”地区概念规划，并实施保护与开发。推进地下空间的规划、建设和管理。启动规划建设市民服务中心、城市综合馆。推进体育公园体育场、国展中心三期、西部交通枢纽等工程，完善新城西区的城市副中心功能。实施文昌路沿线提升行动计划，重点围绕“一线、四片区、六个节点”完善提升文昌路功能。推进瘦西湖世界级公园建设。加快以南河下为重点的民居式客栈建设。实施古运河大王庙以东段等综合整治。组织实施六圩污水处理厂三期和第一水厂扩建项目，开工建设生活垃圾发电厂二期、餐厨废弃物和建筑垃圾综合利用处理厂。新建公交站棚50座，新改建公共厕所119座。

加强城市精细化管理。巩固提升文明城市创建成果，做好迎接全国文明城市公共文明指数测评工作。深入开展“三有三清”活动，加强静态交通建设和管理，运用政策杠杆支持和鼓励公共停车位建设，启动文昌阁地区交通疏解中环线改造工程，实施淮海路苏北医院段、跃进桥交通节点改造。抓好城市主干道绿化亮化美化和背街小巷环境卫生，大力整治违章建筑，做好户外广告、店牌店招和临时疏导点管理。完成二道河、北城河等14条河道的生态清淤。动员社会力量参与智慧城市建设，提高数字化城管水平。完善城区主干道、旅游景点等标识系统。

六、以生态文明为支撑，坚持走绿色发展道路

坚决抓好节能减排。加大节能环保技术、循环利用技术、可再生资源技术以及新型节能建材等新技术、新产品的推广应用。加快环保科技产业园发展。实施节能“八大工程”，抓好重点领域、重点行业的节能减排，坚决淘汰落后产能。加强节水型社会建设，创成各类节水型载体34家，完成节水技改项目10项。完成2家火电厂燃煤机组脱硝改造、7家城镇污水处理厂扩能提标。建立健全节能和污染减排监测、统计、考核三大体系。

推进城乡生态建设。巩固国家森林城市、国家生态市创建成果。提升“五路一环一河”绿化和美化建设水平，推进以瘦西湖、廖家沟、江都水利枢纽等为核心的城市生态园建设，开展全社会立体绿化活动，市区新增城市绿地100万平方米，创建市级园林式单位和居住区20个以上。积极打造南水北调生态廊道，提升大江风光带建设水平。全市新增造林5万亩。深入开展“美好城乡建设行动”，巩固提升农村“四位一体”长效管护成果，疏浚县乡河道210条，整治村庄河塘完成土方1921万立方，新创省三星级“康居乡村”40个、市级“优美乡村”10个。扎实开展秸秆禁烧和综合利用，新增大中型沼气工程30座。

加强资源环境保护。落实土地利用、保护与监管责任。建立土地优供、限供和不供的差别化供应机制。做大做实市、县两级土地储备中心。实施严格的环境准入标准，加强农业面源污染、土壤污染、危险废物污染、重金属污染防治力度，加强对饮用水环境、空气环境的监测监控。继续开展淮河流域、长江流域、南水北调水污染防治工作，确保高邮北澄子河断面水质稳定达标。

七、以民生幸福为根本，不断提高惠民利民水平

着力提高居民收入。继续开展“充分就业示范社区”和“充分转移就业乡镇”创建活动，城镇新增就业5万人，转移农村劳动力1.4万人，培训城乡劳动者2.25万人，实行扬州籍大学生就业年两次推荐制度，职、技校毕业生在扬初始就业率80%以上。安置残疾人就业1000人。推动创业富民，开展创业培训4000人次以上，推介创业项目100个，扶持建设创业孵化基地10个。多渠道增加居民财产性、经营性收入，明晰居民房屋所有权、农民土地使用权，支持居民经营性产权自由流动交易。积极开展富民合作，鼓励和引导居民多样化理财。把建筑业作为富民产业来抓，落实扶持政策，加强市场推介，全年建筑业产值增长15%。完善覆盖城乡的社会保障体系，加快城乡居民养老保险与城镇企业职工养老保险制度转接，全市城乡基本养老保险、基本医疗保险覆盖率均达98%以上。推进被征地农民转参城镇职工基本养老保险。完善社会救助体系，加大困难群体帮扶力度。启动新一轮低收入农户帮扶工程，确保到年底30%的低收入农户人均纯收入达5000元。

推动文化大发展大繁荣。进一步健全公共文化服务体系，增强公益性文化单位服务能力，满足市民对文化的基本需求。加大优秀传统文化的保护和传承，继续推进文化博览城建设，创新文博场馆运营管理机制，支持文博志愿者参与服务。探索在全国具有代表性和示范意义的古城保护模式。牵头做好大运河联合申遗工作。加大非遗项目保护和非遗传承人保障培养力度，培养更多的民间文化能人、文化传承人和群众文化团队带头人，打造特色品牌，活跃社区文化。创作一批现实题材和主旋律作品，打造一批文化艺术精品。加快发展文化产业，完善国有文化企业法人治理结构，创新经营机制，促其做大做强；发展新型文化业态，加快文化产业基地和特色文化产业建设。大力推动创新文化建设，让一切创意想法、一切创新活动得到尊重、受到支持。放大“扬州好人”效应，针对不同群体选树不同典型，不断提高市民法制意识、道德素质和社会文明程度。

加快发展教育、卫生等社会事业。坚持教育优先投入、优质均衡发展。按照就近、优质原则加强幼儿园建设，新（改扩）建公办幼儿园10所，创建省优质幼儿园8所。新创省义务教育现代化示范学校50所。进一步提升高中办学水平。着眼服务全市经济发展，优化提升职业教育质态。推进教育专项资金绩效评价，提高教育经费使用效益，加大对教师再教育、再提高的投入。提升发展医疗卫生事业。进一步优化医疗资源布局。全面实施疾病预防和健康促进工作，倡导市民健康生活方式。加强农村社区卫生服务中心服务能力建设和绩效考核。新农合人均筹资标准提高到350元。支持发展中医药事业。推进人口和家庭公共服务体系建设。办好扬州马拉松赛，推进城市社区“10分钟体育健身圈”建设，不断提高扬州体育人口比例。积极开展地方公共外交。出版《扬州市志（1988-2005）》。

办好民生实事。规范市场价格和收费秩序，保持物价总水平基本稳定。加快区域供水进村入户管网改造。按照确保每户居民有房住原则改进经济适用房保障方式，推动公共租赁住房与廉租住房并轨运行，探索收储社会房源用于住房保障，市区新实现住房保障5650套（户）。全市住房公积金扩面6万人以上。采取有效措施，全面提升小区物业管理水平。继续实施“八老”改造，市区整治老小区95万平方米、公房解危4.5万平方米、改造“城中村”15个。推进农超对接，加强农产品批发市场建设，实施市区农贸市场提档升级新三年规划。公交优先就是市民优先，按照便捷、价廉、舒适的原则，整合市区公交资源，办好城市公交。推进居家养老服务中心、护理员队伍和信息管理系统建设，建立政府为困难老年人购买养老服务制度，城区社区50%的居家养老服务中心达省级示范性标准。加强农村敬老院管理。进一步健全残疾人社会保障和服务体系。

八、以共建共享为目标，切实加强社会管理

深化和谐社区建设。以居民需求为导向，以公益项目为抓手，以多元投入为支撑，构建“三社联动”机制，促进社区、社团、社工之间资源共享、优势互补、协调动作。推进社区网格化管理，用两年时间解决市区全部社区服务用房，提高社区工作人员待遇，市区新建社区服务中心15个。加强和创新社会组织管理，扩大政府购买社会组织服务范围和项目，重点培育发展30个以上社会服务类、公益慈善类社会组织。引导广大居民积极参与社会建设和管理，共同创造幸福美好生活。

推动安全发展。严格落实企业法定代表人安全生产责任制，健全隐患排查治理常态机制，加强消防、交通运输、建筑施工和危险化学品等领域安全工作，推进安全生产标准化和安全生产责任保险。加强食品药品安全监管。完善应急指挥平台和应急信息预警体系，加强应急避难场所、综合应急救援队伍、应急志愿者队伍建设，全面提升应对突发事件综合能力。

维护社会稳定。强化源头治理，巩固“四项排查”工作成果，健全社会稳定风险评估机制。完善人民调解、行政调解、司法调解联动工作机制。开展好各级领导干部下访接访工作。深化法治扬州建设，继续抓好“六五”普法工作。强化流动人口服务管理，全面推行流动人口居住证制度。重视做好民族、宗教工作。深化建设平安扬州，推进技防城市建设，加强虚拟社会管理，积极预防和依法打击各类违法犯罪，不断增强人民群众的安全感。加强国防动员和后备力量建设，创成一批省级双拥模范城（县、区）。

建设为民务实清廉政府

完成今年目标任务，建设人民满意政府，关键在于坚持以人为本、根植人民、执政为民，将人民装在心中；关键在于做到求真务实、狠抓执行，将责任落到实处；关键在于政府要清正、清廉、清明，维护社会公平正义，以好的作风带好的政风、民风。我们要以政府服务的国际化和行政行为的法治化为方向，不断开创政府工作新局面。

改进工作作风，提高服务效能。认真学习贯彻中央关于改进工作作风、密切联系群众的“八项规定”和省委“十项规定”。大力弘扬深入基层调研、尊重群众首创、强化发展实效之风，大力弘扬敢于担当、勇于负责、善于落实之风，大力弘扬定了干、干必成、成必优之风。以科学发展为引领，着力推进制度创新，调适调优责权关系，充分调动市与区（县）、干部与群众各方力量，齐心协力推进经济社会协调发展；以民生改善为目标，深入开展“三下三联三交”活动，一切从维护群众利益出发，一切从群众所急所盼出发，全心全意为群众办实事、办好事、办群众认可和满意的事；以精致高效为导向，优化工作流程设计，强化工作效能考核，深化行政审批制度改革，加强重点工作督查，切实增强政府专业化服务能力。

规范行政行为，坚持依法行政。贯彻落实国务院《关于加强法治政府建设的意见》，切实提高运用法治思维和法治方式深化改革、推动发展、化解矛盾、维护稳定的能力。认真接受市人大的法律监督、工作监督和市政协的民主监督，主动听取各民主党派、工商联、无党派人士和社会各界意见。认真办理人大代表议案、建议和政协提案。严格执行市人大常委会关于讨论、决定重大事项的规定，健全完善重大事项集体决策、专家咨询、群众参与制度，让人民监督权力，让权力在阳光下运行。清理、确认并向社会公告行政执法主体，推行行政执法情况通报制度。推进行政调解和行政指导工作，创新行政复议工作机制，有效化解行政争议。

强化政务诚信，维护政府公信。加强公务员从政伦理和职业道德教育，建立健全政务诚信制度，以政务诚信推动商务诚信，带动全社会诚信建设。扩大政务公开范围，提升行政权力网上运行质量，加强政府新闻发布工作，办好市长信箱、“寄语市长”栏目和12345政府服务热线。推进市、县两级公共资源交易市场建设。所有国资项目招投标实施技术、商务、信用“三合一”评标。强化对政府全口径预算、社会公共资金和节能减排、生态环保等工作的审计与监督。加大失信惩戒力度，坚决纠正政府机关失信行为。

坚持惩防并举，严格廉洁从政。以制度建设为根本，以规范权力运行为重点，着力构建惩治和预防腐败体系，打响“清风扬州”品牌。加强执法监察、效能监察、廉政监察，强化审计监督、舆论监督、群众监督，深入开展民主评议部门、行业风气和基层站所工作。围绕民生幸福工程推进落实情况，深化开展部门“三公开、三报告”活动。加强财政收支管理、专项资金管理和国有资产管理，完善预算管理机制和程序，提高财政资金使用绩效。提倡艰苦奋斗，勤俭办一切事业，打造清廉政府。

各位代表，全面完成今年经济建设和社会发展的各项目标任务，我们责任重大；推进“创新扬州、精致扬州、幸福扬州”和世界名城建设，我们使命光荣。让我们在中共扬州市委的领导下，团结和依靠全市人民，以更加高昂的斗志、更加扎实的举措、更加务实的作风，解放思想，开拓创新，拼搏奋进，为加快建成不含水分的、人民群众认可的、老百姓得实惠的更高水平小康社会而努力奋斗！

打印本页 关闭本页 返回顶部
